# Double-blind controlled dietary cross-over intervention with differentially fertilised intact lettuce leaves shows acute reduction in blood pressure in young adults, associated with faster uptake of nitrate than of phenolics

**DOI:** 10.1007/s00394-022-02961-5

**Published:** 2022-07-23

**Authors:** Othman K. Qadir, Chris J. Seal, Ammar W. Ashor, Michele Tassotti, Pedro Mena, Daniele Del Rio, Mario Siervo, Kirsten Brandt

**Affiliations:** 1 Human Nutrition Research Centre, Population Health Sciences Institute, William Leech Building, Newcastle University, Newcastle upon Tyne, NE2 4HH UK; 2 Food Science and Quality Control Department, College of Agricultural Engineering Sciences, University of Sulaimani, 46001 Sulaymaniah, Kurdistan Region Iraq; 3 Department of Internal Medicine, College of Medicine, Mustansiriyah University, Baghdad, Iraq; 4 Human Nutrition Unit, Department of Food and Drugs, University of Parma, Medical School Building C, Via Volturno, 39, 43125 Parma, Italy; 5 School of Advanced Studies on Food and Nutrition, University of Parma, Parma, Italy; 6 Present Address: School of Life Sciences, Queen’s Medical Centre, Nottingham University, Nottingham, NG7 2UH UK

**Keywords:** Nitrate, Nitrite, Phytochemicals, Young healthy adults, Chlorogenic acid

## Abstract

**Purpose:**

To compare acute effects on blood pressure (BP) of ingestion of visually similar lettuce with controlled high and low content of either nitrate or phenolic compounds.

**Methods:**

In a randomised cross-over design, 19 healthy participants (22–31 years) received 50 g of lettuce containing either 530 mg (8.4 mmol) nitrate + 11 mg (0.03 mmol) phenolic compounds (HNLP); or 3 mg nitrate (0.05 mmol) + 77 mg (0.2 mmol) phenolic compounds (LNHP), obtained by differential fertilisation. Ambulatory BP was recorded along with plasma, salivary and urinary nitrate and nitrite and plasma concentrations of cyclic guanosine monophosphate (cGMP), phenolic metabolites, Trolox equivalent antioxidant capacity (TEAC) and ferric reducing antioxidant power (FRAP).

**Results:**

Compared with LNHP, 3 h post ingestion of HNLP, plasma nitrate increased 0.31 ± (95%CI) 0.12 mM (+ 240%), and salivary nitrate 5.5 ± 1.4 mM (+ 910%); accumulated urinary nitrate excretion increased 188 ± 72 mg (+ 296%) (all *P* < 0.001). Systolic BP was reduced 4.9 ± 4.2 mmHg (*P* = 0.031) between 3 and 6 h after ingestion of HNLP compared with LNHP; systolic BP differences were negatively correlated (*P* = 0.004) with differences in saliva nitrate concentrations. LNHP increased plasma phenolics at 6 h, predominantly 3ʹ-methoxycinnamic acid-4ʹ-glucuronide (ferulic acid-4ʹ-glucuronide), 116%, 204 ± 138 nM more than HNLP (*P* = 0.001); increased cGMP 14% (*P* = 0.019); and reduced FRAP 3.1% (*P* = 0.009).

**Conclusion:**

The acute BP difference within 6 h of consumption matched the plasma/saliva nitrate peak, not the slower changes of plasma phenolics. This is the first double-blind controlled dietary intervention demonstrating differential effects on human physiology by consumption of an intact plant food, where compositional differences were obtained by controlling growing conditions, indicating potential opportunities for health claims relating to precision/vertical farming.

**Clinical trial registration:**

The trial was retrospectively registered on ClinicalTrials.gov, with identifier NCT02701959, on March 8, 2016.

**Supplementary Information:**

The online version contains supplementary material available at 10.1007/s00394-022-02961-5.

## Introduction

Reduced blood pressure (BP) and improved endothelial function have been demonstrated after inorganic nitrate (NO_3_^−^) supplementation in healthy participants and in patients at elevated cardiovascular risk (i.e. hypertension, heart failure) [1]. These positive effects were obtained by the ingestion of inorganic nitrate from different sources including nitrate salts (i.e. sodium or potassium nitrate), or beetroot juice or single batches of leafy vegetables, such as lettuce or spinach.

Dietary inorganic NO_3_^−^ is reduced in vivo by microorganisms in the oral cavity to form nitrite (NO_2_^−^), and then bioactive nitric oxide (NO) in the stomach [2]. NO is also formed endogenously from arginine in blood vessel walls. The NO mediates a key aspect of endothelial function by causing smooth muscle cells to relax, increasing blood flow and reducing BP, by stimulating the activity of soluble guanylate cyclase, forming cyclic guanosine monophosphate (cGMP) [3, 4]. NO is then oxidised to NO_3_^−^. NO_3_^−^ is actively accumulated in the salivary glands to a concentration ~ 10 times higher than in plasma, and excess plasma NO_3_^−^ is excreted in the urine [5].

A few studies indicate that phenolic compounds from plants, including chlorogenic acids, may affect BP in humans [6] in a similar way as dietary NO_3_^−^. For example, phenolic compounds have been hypothesised to improve endothelial function and reduce BP via enhancing the endogenous NO production [7], as a stimulator in endothelium-dependent vasodilation effects mediated by the NO–cGMP pathway. The scarcity of data comparing physiologically relevant intakes of phenolics with NO_3_^−^, may reflect the practical difficulties of this research. Intakes of NO_3_^−^ and phenolics are difficult to distinguish in observational studies, because vegetables are major sources of both these compound types [8]; and in contrast to NO_3_^−^, many phenolic compounds are costly to isolate and difficult to study as pure compounds without substantially changing their bioavailability and probably other properties [9].

Plant genotypes and growing conditions, such as light intensity, temperature and nitrogen fertiliser, are known to cause extensive variability in contents of NO_3_^−^ [10] and (poly)phenols [11]. High concentrations of NO_3_^−^ often occur in beetroots and leafy vegetables, such as spinach, rocket and lettuce, causing concern about potential health risks and legally enforced maximum limits for allowed NO_3_^−^ content, e.g. 200–450 mg/100 g fresh weight for different types of lettuce [12]. However, it is a major challenge for investigations of biological effects of compounds occurring in complex natural (not processed) foods such as fresh vegetables to meet the criteria for double-blind placebo-controlled dietary interventions [13, 14]. This requires products with similar appearance and organoleptic characteristics, whilst differing to an appropriate extent in contents of the relevant phytochemicals. We designed controlled growing conditions to provide reproducible large differences in contents of colourless constituents, NO_3_^−^ and phenolic compounds, whilst the green chlorophylls and yellow carotenoids were less affected, providing similar appearance [15]. The previously published studies that the authors of the present study are aware of, which compared biological effects of entire plant materials differing only in inherent phytochemical composition (not selectively depleted or enriched after harvest), were mostly done in animals, used genetically different plant materials and were not blinded [16–19]. Multiple human intervention studies (reviewed in [1]) have used nitrate-depleted beetroot juice as a placebo to compare with the corresponding nitrate-rich juice; however, this technology is only feasible for liquids such as juice. Recently, substantial interest (scientifically and commercially) has arisen in controlled precision farming systems (‘vertical farming’) [20], which could provide plant foods with optimal inherent composition [21], once science has determined which composition is optimal.

Therefore, this study was designed to investigate if compositional differences between lettuce materials produced under controlled conditions using two different fertiliser treatments [15] could (1) result in corresponding differences in NO_3_^−^ contents in biological fluids (blood, urine and saliva) and of plasma phenolics, and (2) whether any of these outcomes would correlate with BP in healthy subjects, indicating that a causal effect of plant production methods on human physiology could be feasible.

One of the fertiliser treatments provided lettuce material with controlled high NO_3_^−^ and low phenolics contents (HNLP), whilst the other contained low NO_3_^−^ and high phenolics (LNHP). A key feature of this intervention was that the genetically identical LNHP and HNLP lettuce materials were sufficiently similar in appearance, colour and taste to make blinding feasible to the participants and investigators. This design thus allowed assessment of differences in effects of plant materials on human physiology that were caused solely by the plants’ growing conditions (fertiliser).

Referring to the anticipated BP-lowering effects of both NO_3_^−^ and phenolics, we hypothesised that a relative drop in BP in the treatment with a high concentration of a phytochemical in the lettuce, during the time interval where the plasma concentration of this phytochemical peaked, would indicate a potential BP-lowering effect of that phytochemical. So, differences in timing of the maximal concentrations would allow each lettuce treatment to act as control for the other treatment during different time ranges. Metabolites (NO_3_^−^, NO_2_^−^ and phenolics) were measured in biological fluids to define when their concentrations peaked, and other markers (cGMP, FRAP and TEAC) have been reported as informative in relation to mechanisms of effects on BP.

## Materials and methods

### Participants

Twenty-four healthy participants were recruited, with twenty completing. Participants attended the NU-Food research facilities at Newcastle University from 25 February 2015 until 04 August 2015. Four participants withdrew from the study due to other commitments, after the recruitment but before the first intervention day, and data from one participant were excluded due to non-compliance; so the final analysis included the data from the 19 completing and complying participants. The trial was approved by the Ethics Committee of Faculty of Science, Agriculture and Engineering at Newcastle University (Trial ID: 15-QAD-15). The trial was registered on ClinicalTrials.gov with identifier NCT02701959. Due to an administrative error at Newcastle University, the registration was not recorded until after the intervention had taken place. The CONSORT checklist is Table S1 in the Online Supplementary Material.

### Inclusion criteria

Age ≥ 18 years; Willingness to comply with dietary restrictions and consume intervention foods; sufficient knowledge of English to understand study material.

### Exclusion criteria

Current participation in other clinical investigations; mouthwash users; vegetarianism (implying atypical habitual nitrate intake); use of antihypertensive medication; history of cholesterol lowering medication; history of cardiovascular or peripheral vascular disease; history of any major illness such as cancer; a psychiatric illness; recent history of asthma, renal, liver or gastrointestinal disease; use of antibiotics within previous 2 months; current or recent (within previous 6 months) significant weight loss or gain (> 6% of body weight); woman who were pregnant, lactating or wishing to become pregnant during the study; previous diagnosis of type 1 or type-2 diabetes treated with insulin; major surgical operations, which may interfere with the study outcomes; alcohol intake > 21 units/week for men and > 14 units/week women.

### Study design and randomisation

This was a cross-over, double-blind, randomised controlled intervention trial. Treatment sequence was allocated to each participant using the random number generator in Microsoft Excel. In the evening before the study day, 50-g portions of lettuce material for the two interventions (HNLP and LNHP) were harvested and placed in opaque bags. A staff member outside the study team then randomised the bags and labelled them with the participant numbers, blinding the treatment to both the researcher and the participant. The two interventions had similar organoleptic characteristics and presentation, including identical colour and volume (Photo as Fig. S1 in the Online Supplementary Material).

### Interventions

Participants received a 50-g lettuce portion of HNLP (~ 530 mg NO_3_^−^ and ~ 11 mg phenolic compounds per portion) or LNHP (~ 3 mg NO_3_^−^ and ~ 77 mg phenolics) in the morning on two separate days. The growing conditions and detailed composition of the HNLP and LNHP lettuce materials have been described elsewhere [15].

### Study protocol

**Fig. 1:**
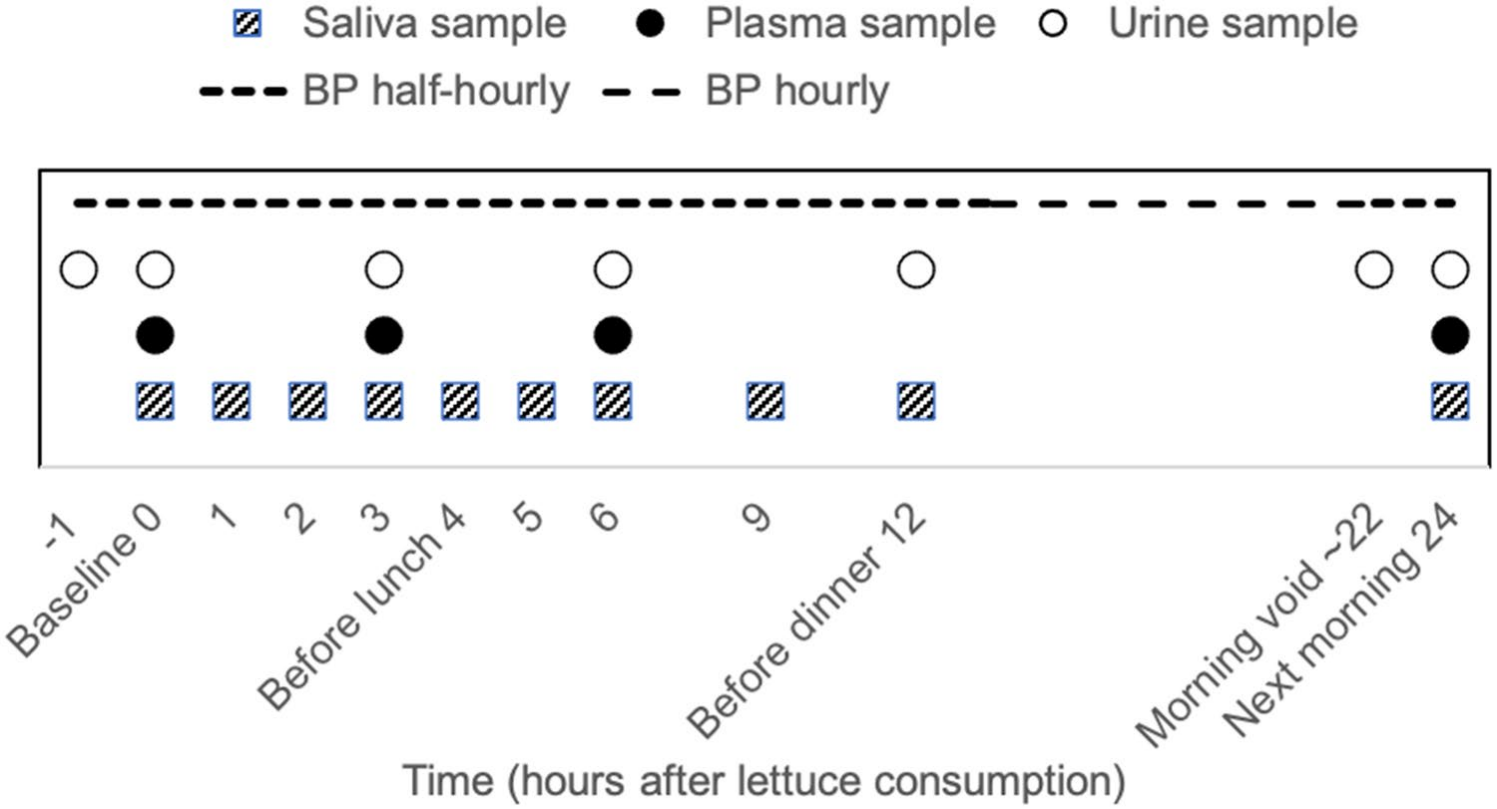
The design of each session in the intervention trial. At least 3 weeks between the two sessions; cross-over, with randomised, double-blind sequence of treatments; restrictions on nitrate-rich foods for 24 h before each session, and no mouthwash throughout the study period. Automated BP measurements from before lettuce consumption until 24 h after. Standard meals consumed 12 h before, 4 h after and 12 h after lettuce consumption. Samples at − 1, 9, 12 and approx. 22 h collected by the participants at home, all other samples collected at the research facility

The study comprised a screening visit to evaluate the eligibility for the study and two intervention sessions, separated by a 3-week washout period to avoid carry-over effects. Body weight, height and waist circumference were measured according to standardised protocols. A body composition analyser TANITA BC420 MA was used to measure fat mass. Body mass index (BMI) was calculated as weight (kg)/height squared (m^2^). Eligible participants were asked to refrain from using mouthwash throughout the entire study period, and to follow a low nitrate diet from 24 h prior to the start of each intervention session, excluding high NO_3_^−^ foods: rocket, spinach, other leafy vegetables, radish, beetroot, cured meat, cured seafood, cured fish and mature cheese. To minimise random variation in diet, for each intervention, participants were provided with two portions of a standard meal, the Chicken Hotpot (372 g; ASDA Stores Ltd., Leeds, UK), to consume at home the evening before and the evening after the lettuce intervention, and a Mediterranean Vegetable Penne Pasta, Birds Eye Ltd., London, UK (350 g) for lunch on the intervention day. The NO_3_^−^ content of these meals was measured as 55.26 and 61.07 mg/meal, respectively. They were also provided with low-NO_3_^−^ mineral water (NO_3_^−^ < 0.1 mg/L, Buxton Mineral Water, Nestlé Waters UK, Buxton, Derbyshire, UK) to consume from the evening before the study visits until the end of the study visit (~ 36 h) and asked to fast for at least 12 h before the study visits. The International Physical Activity Questionnaire (IPAQ) was used for assessment of physical activity level [22], energy intake was assessed using the EPIC Food Frequency Questionnaire (FFQ) [23]. All measurements were performed at the start of each intervention period (HNLP or LNHP) with the exception of the FFQ, which was conducted only at the first session. Urine, saliva and blood samples were collected throughout both visits in each session, from before the consumption of the intervention meal and with the last sample 24 h post-consumption (Fig. 1).

### Blood pressure measurements

Baseline BP was measured in triplicate with a Mobil-O-Graph NG (Germany). Participants were then fitted with a Mobil-O-Graph NG (I.E.M. GmbH, Germany) to monitor 24-h systolic and diastolic BP. Ambulatory Blood Pressure Monitoring (ABPM) measurements were started when lettuce consumption started, and automatically taken every 30 min during day time (between 0900 and 2200 h) and every 60 min at night (between 2200 and 0900 h) to minimise the potential impact on sleep quality. The device was approved by the British Hypertension Society and was validated prior to use. Participants were instructed on how to operate the device, to stop moving (e.g. sit down) when a measurement started and to otherwise continue their normal activity during the monitoring period. Out of 1537 attempted measurements, 67 failed, mostly due to movement. Values for systolic BP higher than 160 or lower than 70 were disregarded (6 occurrences). Missing or disregarded values were replaced with interpolated values (average of the nearest values before and after the aberrant measurement) to calculate averages for each time point.

### Urine collection

One urine sample (first morning void) was collected by the participants at home before arriving for the experimental day. At NU-Food research facility, one sample was collected at baseline (just before consuming the lettuce) and further urine samples (all that the volunteer could produce) were collected at set times after the supplementation, one for the interval up to 3 h and the next from 3 to 6 h. Subsequent collections took place at home using two plastic containers, one for all urine produced between 6 and 12 h and another for 12 to approx. 23 h after supplementation (which included the subsequent morning void). Samples for each time period were weighed to determine volume and aliquots stored at − 20 ℃ until analysis.

### Saliva collection

Small saliva samples (~ 2 mL) were collected in disposable plastic containers at baseline and then every hour for 6 h at the NU-Food research facility and immediately transferred to − 20 ℃; additional saliva samples were collected by the participant at 9 and 12 h post intervention at home (kept in the freezer at home) with a final sample taken at 24 h.

### Plasma collection

Venous blood samples were collected in lithium-heparin containing tubes (~ 6 mL) at baseline and then 3, 6 and 24 h after the consumption of the lettuce. Samples were centrifuged within 30 min at 3000 rpm for 10 min at 4 ℃ and plasma was immediately transferred to − 80 ℃.

### NO_3_^−^ and NO_2_^−^ analysis

NO_3_^−^ concentration in plasma, saliva and urine, and NO_2_^−^ in saliva and urine were analysed by GC–MS as described in [24], based on [25]. Plasma was not treated to preserve NO_2_^−^, and this compound was not reliably detected when the samples were analysed, so no data on plasma NO_2_^−^ are presented.

### Phenolic compound analysis

A range of phenolic compounds, consisting of various caffeoylquinic acid-derived metabolites (5-caffeoylquinic acid, 4ʹ-hydroxycinnamic acid-3ʹ-glucuronide (*aka* caffeic acid-3′-glucuronide), 4ʹ-hydroxy-3ʹ-methoxycinnamic acid (*aka* ferulic acid), 3ʹ-methoxycinnamic acid-4ʹ-glucuronide (*aka* ferulic acid-4′-glucuronide), 3ʹ-methoxycinnamic acid-4ʹ-sulphate (*aka* ferulic acid-4′-sulphate), 3ʹ-methoxycinnamic acid-4ʹ-glycine (*aka* feruloylglycine), 4ʹ-methoxycinnamic acid-3ʹ-glucuronide (*aka* isoferulic acid-3′-glucuronide), 3-(3ʹ-methoxyphenyl)propanoic acid-4ʹ-sulphate (*aka* dihydroferulic acid-4′-sulphate), and 3-(4ʹ-hydroxyphenyl)propanoic acid-3ʹ-sulphate (*aka* dihydrocaffeic acid-3′-sulphate)) were identified and quantified in the plasma samples using an UHPLC-QqQ-MS/MS analysis as described in [26]; the retention times found and corresponding optimised SRM conditions are shown on Table S2 in the Online Supplementary Material. The compounds are named according to recent recommendations for standardising nomenclature for phenolic catabolites [27]. Six samples (out of 152) showed no peaks at all, presumably caused by an error during sample preparation, handling, or analysis, so these values were excluded from the data analysis. Two of these 6 erroneous samples were before start of lettuce consumption (time = 0), those were replaced with the corresponding baseline values for the same participant before the start of the other treatment. For other samples, where some peaks were missing, but not all, those individual missing peaks were analysed with the value 0.

### TEAC and FRAP assay

Plasma TEAC and FRAP were measured almost as in [28], using an ABX Pentra 400 (Horiba Medical, Northampton, UK), except that the wavelengths used for measurements were 420 nm for TEAC and 593 nm for FRAP, respectively. Two out of the 152 samples gave a value of 0, these data were treated as missing values rather than 0s.

### cGMP assay

cGMP in plasma was determined using the immunoassay ELISA kit ADI-900-013 per the manufacturer’s instructions (Enzo Life Sciences, Inc., USA). Samples were non-acetylated as concentrations greater than 1 pmol/mL were expected [29]. Assays were run in duplicate. Values were missing for 5 out of the 152 samples. Three of these 5 erroneous samples were collected before start of lettuce consumption (time = 0), those were replaced with the corresponding baseline values for the same participant before the start of the other treatment.

### Statistical analysis

A sample size of 20 with 80% power was calculated based on [30], where a meal of spinach (220 mg of nitrate) reduced systolic BP by 6.4 mmHg (SD 6.9) after 180 min in healthy adults (age 38–69 years), compared with a low nitrate rice meal, as the published study most similar to this project. To accommodate expected attrition, a total of 24 participants were recruited.

Twenty participants completed the study. During data analysis, it was discovered that one person excreted extremely large amounts of urinary nitrate, > 200% of NO_3_^−^ intake from study foods during the recording period, demonstrating either non-compliance with diet restrictions or some pathology causing excessive endogenous NO_3_^−^ formation, so all data from this person were excluded due to suspected non-compliance. This exclusion affected the values of the outcomes (in particular average urinary NO_3_^−^ excretion) but it did not affect any key conclusions, since generally the same *P* values were significant with and without this participant’s data included.

The data analysis was not pre-specified, since this was an explorative study. The start dates for the individual interventions were spread out over 4 months, much longer than the 3-week washout period, which again was much longer than the one day intervention periods, so period/sequence effects were not included as a factor. However, the tests were robust regarding outliers, by including data from several time points in every comparison and presenting 95% CI intervals where feasible.

Blood pressure data were used for 2 different comparisons: 1. As difference between treatments for each 3-h period, using paired 2-sided *t* tests comparing the averages for each participant of all measurements in each treatment session during that period (both systolic and diastolic BP), to obtain an appropriate time resolution; 2. As average difference in systolic BP between the two treatments during the periods of 1, 3 or 12 h corresponding to saliva samples taken at the end of that period, and then correlated with the corresponding difference in saliva NO_3_^−^ for the same person and time period, to test for a main effect of bioavailable NO_3_^−^. Data from saliva samples were used for this, rather than plasma, due to the higher frequency of sampling providing more data points.

For all other variables, since they were measured at baseline (immediately before lettuce consumption), analyses of main effects (difference between treatments in overall effect, across all time points) were done separately on all recorded data points and on the change from baseline (subtracting the baseline value from all other data recorded for the same participant in the same session), to compensate for potential confounders affecting the participant, the session or both. For most variables, the two analyses resulted in the same conclusion (the difference between treatments was either significant or not significant), so only the results for all data points are shown. For plasma phenolics and cGMP, only the change from baseline showed significant differences between the main effects of treatments; therefore, the results for the change from baseline are shown alongside with the results using all data points.

Outcomes were expressed as mean ± 95% CI unless otherwise specified; for outcomes where the effects of treatments were much larger than the sum of the 95% CIs, no statistical analysis was performed. *P* < 0.05 was considered statistically significant. Concentrations of NO_3_^−^ and NO_2_^−^ were analysed using logarithmic transformation, to ensure homogeneous standard variance at each time point, and therefore these data are presented using log scales in the figures. Data analyses were done using Microsoft Excel and Minitab^®^.

## Results

**Fig. 2:**
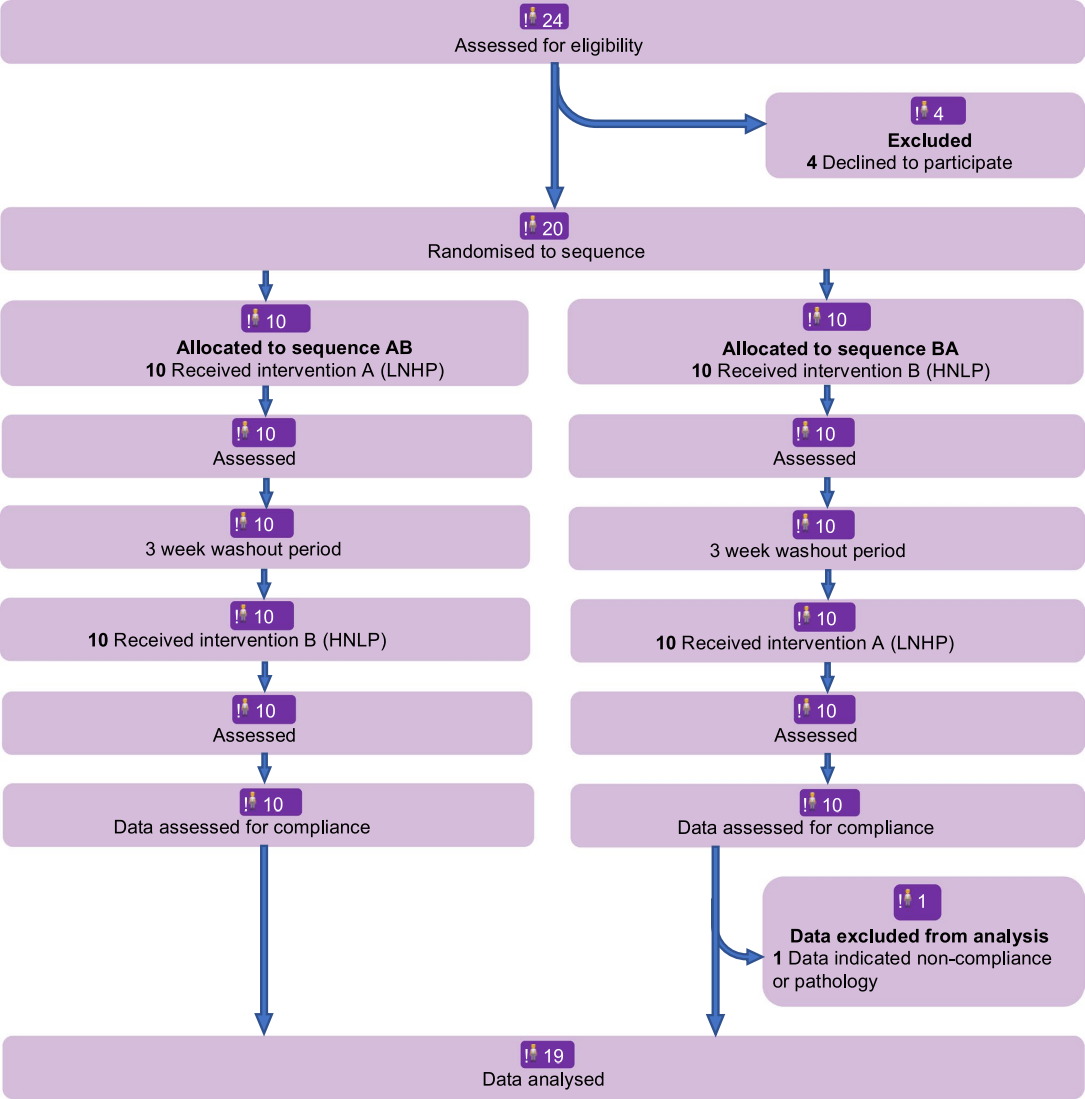
CONSORT flow chart for the cross-over design. The participant whose data were excluded consistently excreted substantially more NO_3_^−^ in the urine than was provided in the intervention and other foods

The baseline characteristics of the 19 participants whose data were included in the analysis are shown in Table 1, and the CONSORT flow chart as Fig. 2. For each characteristic, the mean, SD and range fully conform to the expected values for the intended population (young healthy adults).

**Table 1:** Baseline characteristics of the participants^a^ before consumption of lettuce

Characteristic	Mean	Standard Deviation	Range
Age (y)	25.0	2.9	21–30
Height (cm)	168	8.6	150–181
Body weight (kg)	62.1	7.7	53–80
Body mass index (kg/m^2^)	22.0	1.6	20–25
Waist circumference (cm)	80.3	5.2	71–92
Fat mass (%)	19.8	5.7	9–28
Heart rate (bpm)	68.8	9.9	44–86
Systolic BP (mmHg)	111	10	93–134
Diastolic BP (mmHg)	72.0	8.8	56–85
Energy intake (kcal/d)	2628	1051	1073–4941
Physical activity (MET^b^s)	3.4	2.1	1.0–7.7

^a^*N* = 19, 11 females and 8 males

^**b**^*MET* Metabolic equivalent of task

**Fig. 3:**
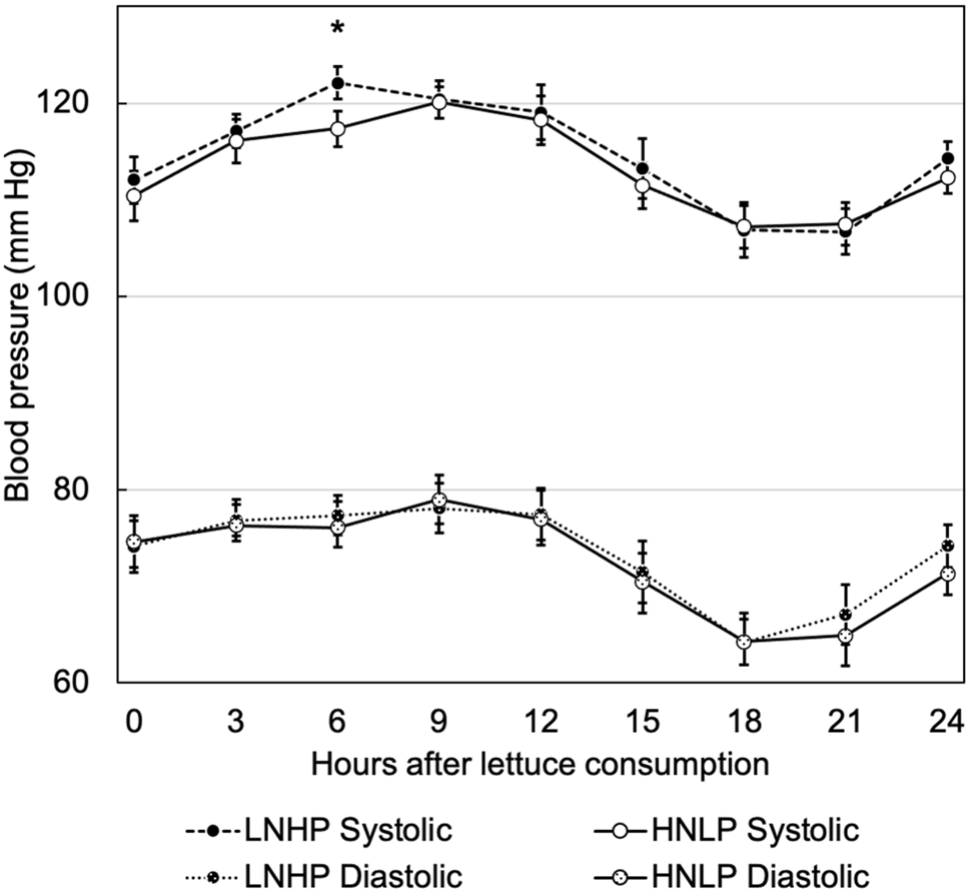
The effect of HNLP and LNHP lettuce consumption on 24-h ABPM systolic and diastolic BP. Average values for each 3-h interval (values recorded up to 3 h before) are shown as circles, SEM are shown as error bars, *Indicates a significant difference (*P* = 0.031). *N* = 19, each data point consisting of an average of 3–6 measurements (less frequent during the night)

An overview of the blood pressure data is provided in Fig. 3, showing the well-known diurnal variations, and a significant difference was observed between 3 and 6 h after ingestion. More detailed overviews are found in Suppl. Material Fig. S2 and S3, showing 95% CIs and hourly values, respectively.

**Fig. 4:**
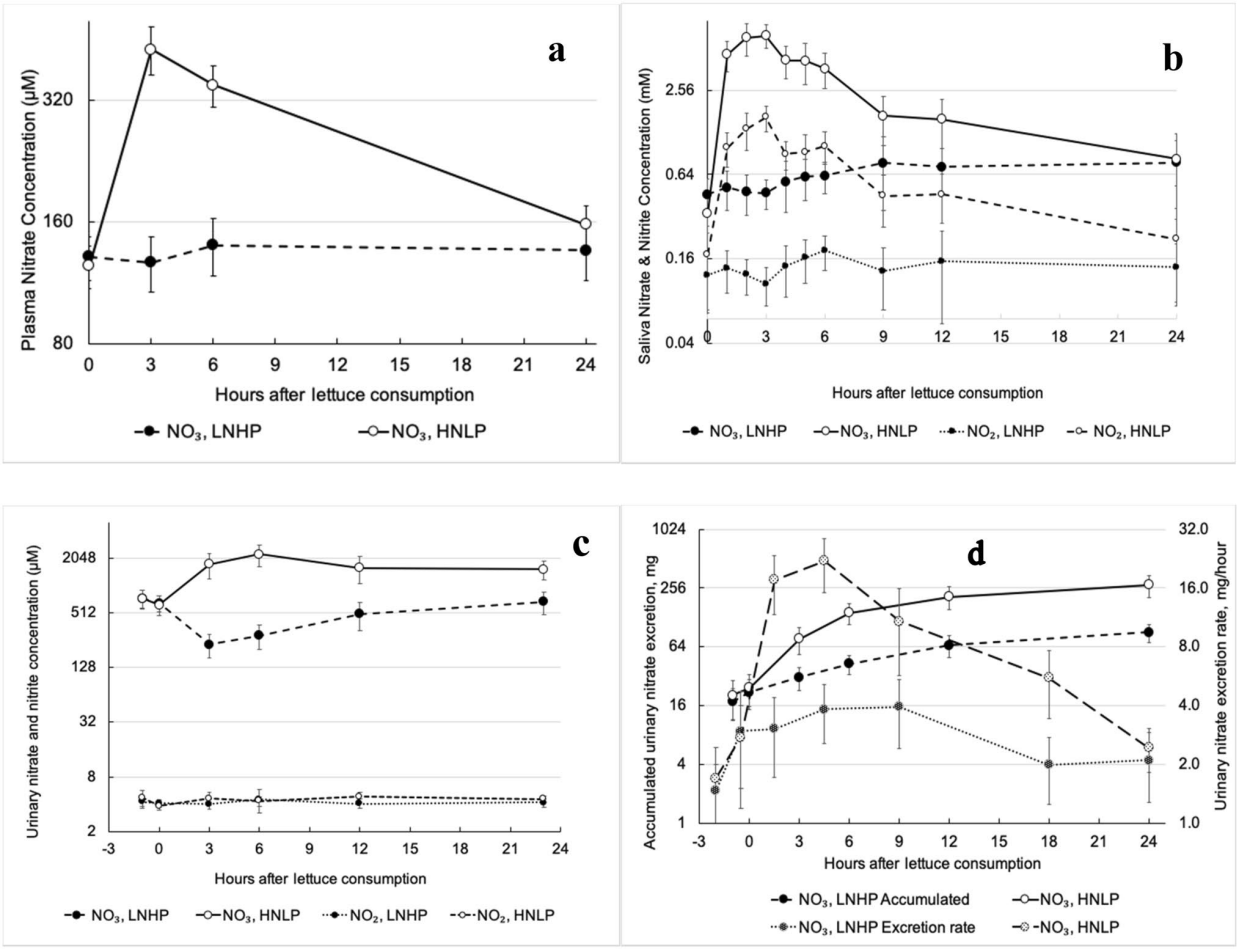
The effect of HNLP and LNHP lettuce consumption on the concentrations of **a** plasma NO_3_^−^, **b** salivary NO_3_^−^ and NO_2_^−^, **c** urinary NO_3_^−^ and NO_2_^−^, all in µM. Urinary NO_3_^−^ excretion (**d**) is in mg/h and accumulated excretion in mg. Data are all shown on log scales, as mean ± 95% CI, with *N* = 19

Plasma NO_3_^−^ and salivary NO_3_^−^ and NO_2_^−^ concentrations increased substantially after ingestion of HNLP lettuce, peaking at around 3 h, but remained unchanged after consumption of LNHP (Fig. 4a, b, respectively). In saliva, the NO_3_^−^ concentration appeared to peak slightly earlier than for NO_2_^−^ and decline faster after the peak, with NO_2_^−^ still noticeably elevated at 12 h, although all of these returned to baseline levels after 24 h.

Urinary NO_3_^−^ concentration increased 3 and 6 h after ingestion of HNLP, but decreased during the same period after consumption of the LNHP (*P* < 0.001). Both almost returned to the initial values after 24 h. Urinary NO_2_^−^ concentrations were very low and showed no significant changes or differences (Fig. 4c).

The urinary NO_3_^−^ excretion rate increased in the HNLP treatment until 24 h after consumption, whilst it remained almost unchanged with LNHP (Fig. 4d). During 24 h after lettuce consumption, the total accumulated urinary NO_3_^−^ excretion was 264 ± 72 mg in the HNLP treatment and 78 ± 20 mg for LNHP, which should be compared with the 646 and 119 mg NO_3_^−^, respectively, provided in food (meals + lettuce) in each treatment during the same period.

**Fig. 5:**
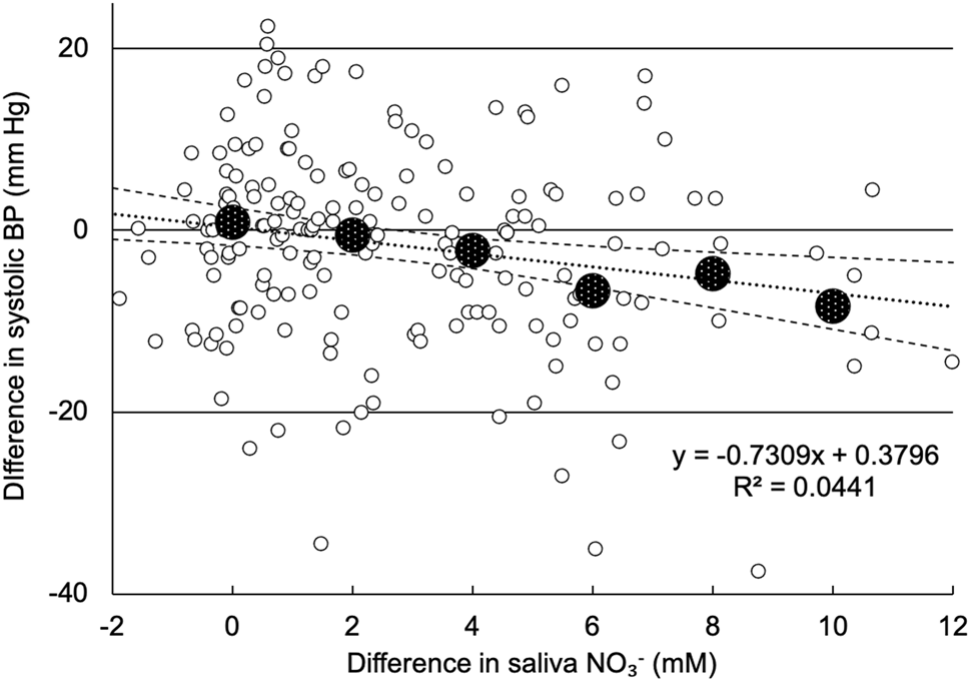
Differences between values after HNLP and LNHP lettuce consumption for salivary NO_3_^−^ and corresponding values for systolic blood pressure, as an average value for each person during each time period where saliva samples were collected. Small white circles are individual measurements (*n* = 190, with 10 values from each person), large patterned circles show the average BP difference for each 2-mM range of salivary NO_3_^−^-values. The dotted line is the regression trend line with formula and *R*^2^ value (with *P* = 0.0036), the two dashed lines show the 95% CI corresponding to the trend

To compensate for the confounding by the substantial diurnal changes as well as large individual differences in response to the treatment, the values for the difference between treatments in systolic BP were plotted against the difference in salivary NO_3_^−^ concentration for each person and each time period, resulting in the regression in Fig. 5. It shows that in persons and at times when the salivary NO_3_^−^ increased by > 6 mM in the HNLP treatment compared with LNHP, corresponding to the average increase at 2 and 3 h after ingestion (Fig. 4b), on average the systolic BP fell by 6.5 ± 4.4 mm. In contrast, the BP did not change when there was no difference in salivary NO_3_^−^ concentration between the treatments.

**Fig. 6:**
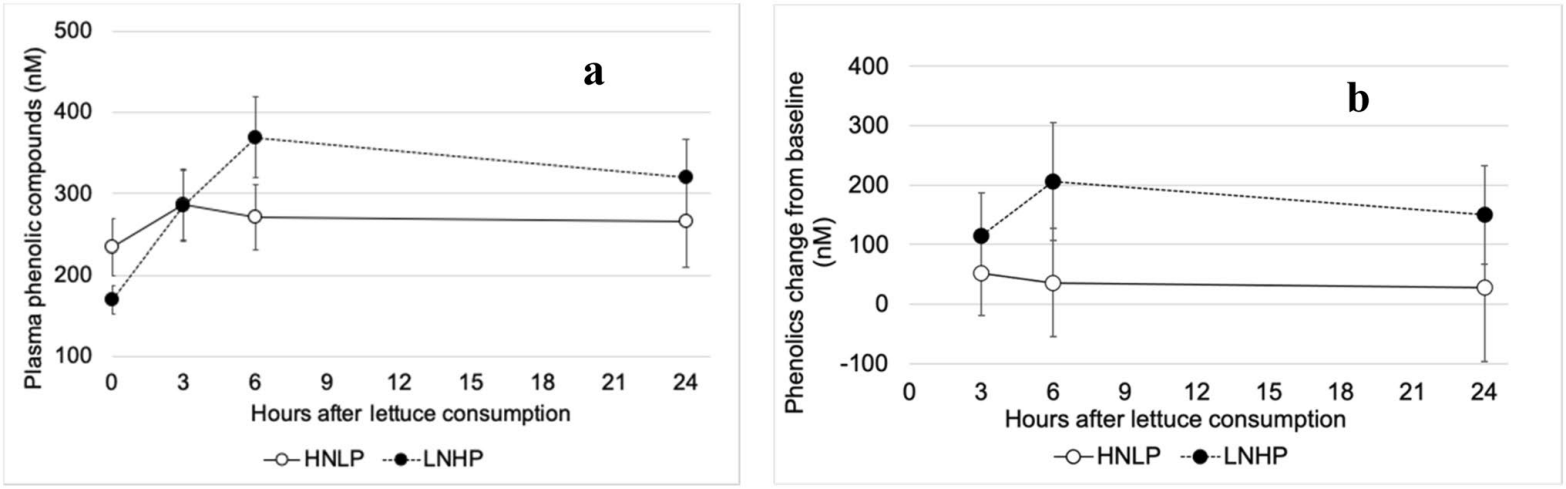
The effect of HNLP and LNHP lettuce consumption on plasma concentrations of the sum of all measured phenolic compounds. **a** shows absolute values, **b** changes from baseline. Data expressed as mean ± 95% CI, *N* = 19

Nine phenolic metabolites were detected in plasma; all were hydroxycinnamic acids and their derivatives. The measured concentrations of plasma phenolic compounds were very variable, both between and within participants, partially obscuring general effects of treatments. Still, a substantial increase (*P* = 0.001) was observed for the sum of all phenolic compounds after ingestion of LNHP lettuce, with highest value 6 h after ingestion, this increase did not return to baseline by 24 h, whilst the HNLP treatment only caused a transient insignificant increase in plasma concentrations (Fig. 6). The most abundant compound detected was 3ʹ-methoxycinnamic acid-4ʹ-glucuronide (Suppl. Material Fig. S2a), which showed a similar pattern as the sum of all the other phenolic compounds (Suppl. Material Fig. S2b), although none of the other compounds showed significant treatment effects individually.

**Fig. 7:**
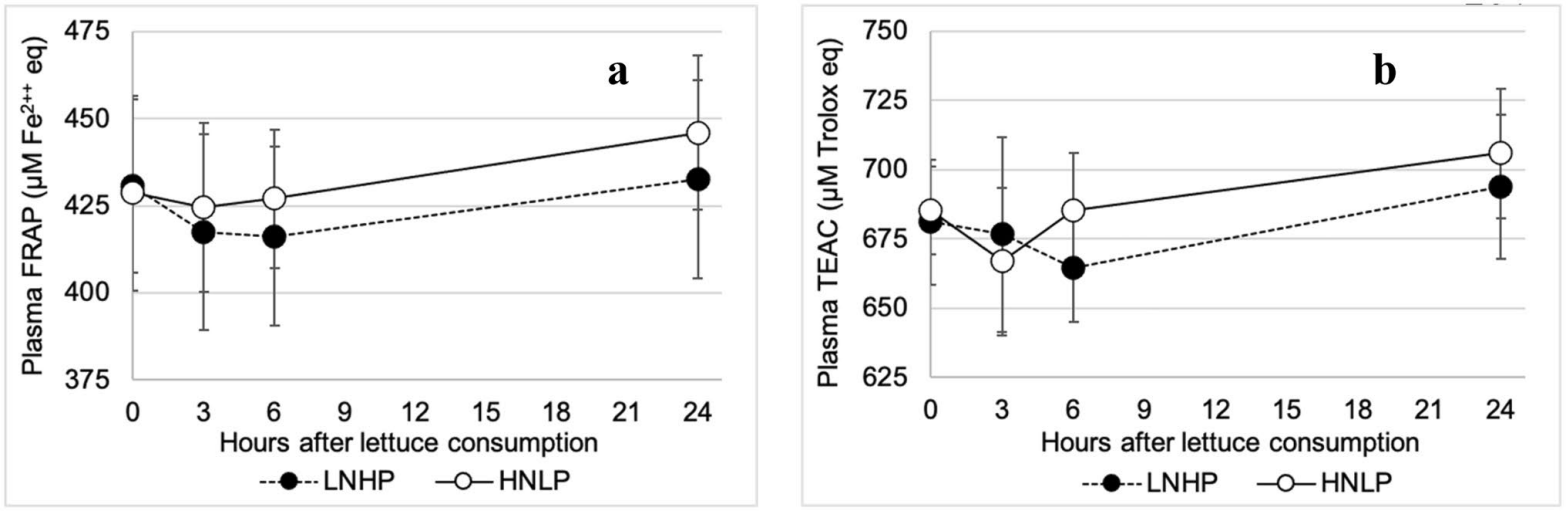
The effect of HNLP and LNHP lettuce consumption on **a** FRAP (ferric reducing antioxidant power) and **b** TEAC (Trolox equivalent antioxidant capacity). Data expressed as mean ± 95% CI

Both FRAP and TEAC showed significant effects of time after lettuce consumption, the values fell and then rose during the intervention period, *P* = 0.001 and *P* = 0.007, respectively (Fig. 7a and b). For FRAP, the values became lower for the HNLP, resulting in a significant main effect of treatment (*P* = 0.009, Fig. 7a), whilst no significant differences in main effect of treatment were observed between HNLP and LNHP for TEAC (*P* = 0.27, Fig. 7b).

**Fig. 8:**
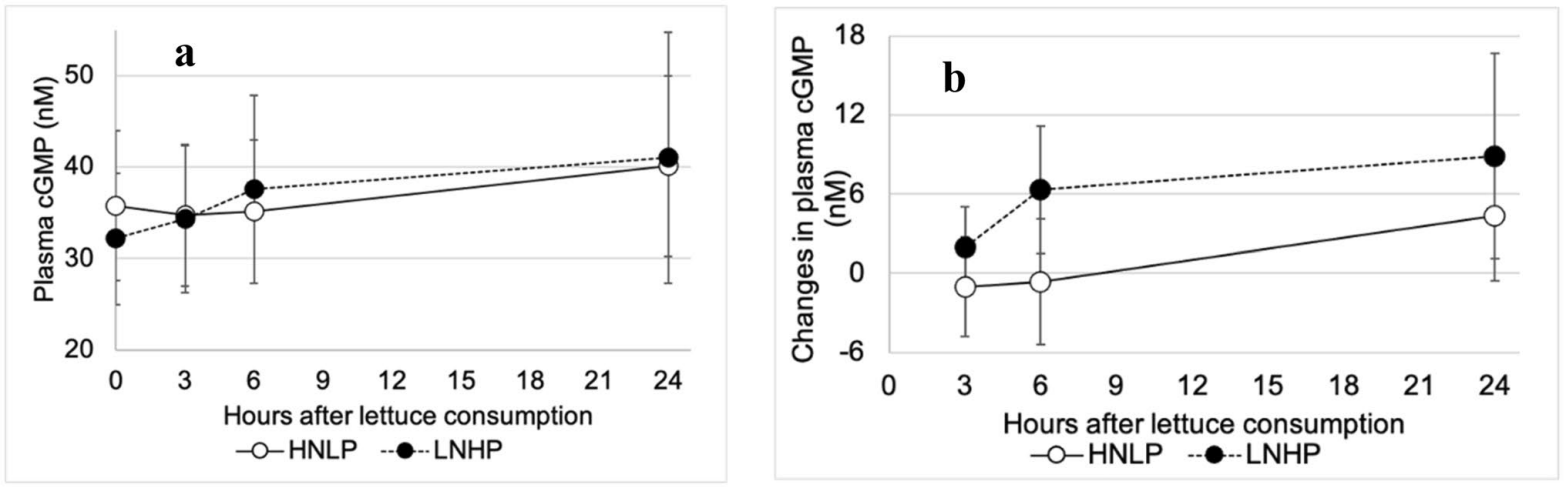
The effect of HNLP and LNHP lettuce consumption on the plasma concentration of cGMP, cyclic guanosine monophosphate. **a** shows absolute values, **b** changes from baseline. Data expressed as mean ± 95% CI

For cyclic guanosine monophosphate (cGMP), no significant differences in the absolute values were observed between HNLP and LNHP (*P* = 0.98), Fig. 8a. However, across all post-consumption time points, the change from baseline values (Fig. 8b) was substantially larger in the LNHP treatment than HNLP (*P* = 0.019).

## Discussion

### Relevance for measuring the acute effect of lettuce consumption on BP in young healthy adults

The present study found a small, marginally significant (P = 0.038) difference in BP between treatments during the period of maximum plasma/saliva NO_3_^−^ concentrations in the HNLP treatment, and a more significant overall correlation between increased NO_3_^−^ concentration in saliva and reduced systolic BP. These results thus supported the hypothesis that the vegetable-derived NO_3_^−^ intake resulted in the lowering of the BP. The magnitude of the effect was within the range found in non-blinded studies of, e.g. spinach [30, 31] in similar populations. However, this is the first double-blind study to use intact plant material with the same genotype and similar appearance, differing in composition only due to different growing conditions (fertiliser), as the treatments compared in an acute human intervention trial.

### Relevance regarding the roles of dietary NO_3_^−^ and phenolics in plasma concentrations of these compounds and their metabolites

The present results demonstrated that one portion of high-nitrate low-phenolics HNLP lettuce material containing ~ 530 mg of NO_3_^−^ more than doubled the concentrations of plasma, salivary and urinary NO_3_^−^ and salivary NO_2_^−^, within the first 3 h of consumption. In contrast, the low-nitrate high-phenolics LNHP material containing ~ 71 mg soluble phenolic compounds increased the plasma content of 3ʹ-methoxycinnamic acid-4ʹ-glucuronide and overall phenolic metabolites significantly, but slower than the NO_3_^−^, with the highest concentration at 6 h, as expected from other studies [26]. There was little or no increase in plasma NO_3_^−^ (nor excretion into saliva or urine) in treatments and time points with high concentrations of phenolic compounds, and the observed increases in NO_3_^−^ and NO_2_^−^ could be fully accounted for by the NO_2_^−^ in the HNLP lettuce: The HNLP provided 8.5 mg NO_3_^−^/kg body weight (vs 0.04 mg/kg body weight in the LNHP), a similar dose to the 6.5 mg/kg body weight beetroot juice supplement dose used by Miller et al. [32] who found similar levels and time course of plasma NO_3_^−^ in a study with older adults. Dietary NO_3_^−^ returned to a baseline of plasma and saliva NO_3_^−^ and NO_2_^−^ within 24 h after a single dose, as also previously observed [33, 34]. Our results are also in accordance with ~ 25% of ingested NO_3_^−^ entering the entero-salivary circulation through the plasma into the saliva, and being reduced to NO_2_^−^ by bacteria on the tongue [5, 35, 36].

Taken together, these results demonstrate that the increase of the NO circulation through the entero-salivary circulation via NO_3_^−^-NO_2_^−^-NO pathway could be caused by dietary NO_3_^−^ [2], but not by the phenolic acids [6, 7] found in the same type of food.

### Relevance regarding urinary excretion of dietary NO_3_^−^

The urinary excretion of NO_3_^−^ in the HNLP treatment peaked in the interval between 3 and 6 h and returned to the baseline by 24 h (Fig. 4d), similarly as in saliva (Fig. 4b), as would be expected if both saliva and urine reflect the concentrations in plasma, and confirming that dietary NO_3_^−^ is highly bioavailable. However, the total amounts of 264 and 78 mg excreted in urine within 24 h after consumption of the HNLP and LNHP lettuce, respectively, were much less than the total dietary intakes of 646 and 119 mg, respectively, from lettuce, lunch and dinner during this period, plus an unknown contribution from endogenous synthesis of NO_3_^−^ via L-arginine and NO [37]. This corresponds to data from the 1980s in rats [38] and humans [39]; however, it is still not known whether these discrepancies are caused by dietary NO_3_^−^ suppressing the endogenous synthesis, and/or if all of the remaining 382 mg NO_3_^−^ from the lettuce was lost via other routes, e.g. excreted in faeces or via sweat, exhaled via the air, etc. [40], or metabolised to nitrogen or protein by the gut bacteria [39]. Slight modifications of the present experimental design could be used to address those questions, such as using stable isotopes of N and O for the nitrate fertiliser used to produce the HNLP lettuce; and collecting and analysing samples of faeces and exhaled air, as well as urine, plasma and saliva.

### Relevance for practical implementation to improve population health

Despite known beneficial associations of cardiovascular health with long-term intake of leafy vegetables (the primary contributor to dietary NO_3_^−^ intake, although confounded with intake of phenolic compounds) [8], experimental studies of this relationship have tended to be short-term, less than 70 days [1]. However, whilst useful for elucidation and/or confirmation of mechanisms, short-term studies cannot predict long-term effects, and studies using enriched supplements cannot predict effects of actual foods [41]. The health benefits of increased vegetable consumption are well recognised, and a causal role of NO_3_^−^ is scientifically plausible [37, 42, 43]. However, in the absence of evidence from long-term food-based studies, which would require (until now unavailable) affordable supplies of vegetables with consistently defined NO_3_^−^ contents, most food producers and authorities still consider NO_3_^−^ a harmful ‘contaminant’ [12, 44], engaging R&D primarily to minimise its content [20, 21]. The experiences from the present study could however be combined with recent advances in precision farming [20] to enable provision of the required evidence for scientifically evidenced improvements in plant food composition.

### Limitations of the study

Since the trial compared the two different lettuce types, without a negative control treatment, it is possible that the LNHP treatment on its own would also have reduced BP, possibly by a different mechanism, in which case the BP reduction values measured for HNLP lettuce are actually underestimated. It is an inherent feature of the method used for controlling the nitrate content (fertilisation intensity) that it also affects accumulation of phenolics (and other plant constituents) [15]. This is a drawback in relation to its use as an experimental tool (as in the present study). However, valuable regarding that this reflects the differences amongst production methods used commercially [45]. Future studies may address this drawback through relatively limited additional development; for example, using a placebo-controlled chlorogenic acid supplement [6, 7] together with the low phenolic plant phenotype, to equalise the phenolic intake in the treatments (this high–high treatment could be a third arm), or use a genotype impaired in chlorogenic acid biosynthesis, either a natural mutant [16] or created through genetic technology, to obtain a low–low treatment.

The lowering of BP was observed in young healthy adult participants, a population where reduced BP is not as such a health benefit. The results are not necessarily relevant for people with hypertension, whose BP regulation does not function normally, and who may not be affected in the same way [46]. Relevant potential benefits of NO_3_^−^ ingestion in a young population may relate to oral health or some types of cognitive or athletic performance which may be enhanced by increased blood flow.

The ABPM data were extremely variable, on average differing 8 mmHg from one reading to the next, with approx. 10% of sequential differences > 20 mmHg, indicating that some of the measurements were inaccurate. Improved equipment or procedures (e.g. to relax 5 min before a reading) would have increased the power of the study to detect effects on BP.

The postprandial blood samples were only collected every three hours, not every hour as for the saliva samples, limiting the detail obtained in the time courses of the metabolites. On the other hand, the results highlight the usefulness of saliva or urine samples to monitor the NO_3_^−^ status non-invasively, particularly in contexts where phlebotomy is difficult, for example studies involving children or community surveys.

The results of the TEAC, FRAP and cGMP measurements did not clearly support or contradict any well-known hypotheses. However, not reporting them would constitute a publication bias, since the data might be useful for future meta-analyses.

## Conclusion

This trial showed that lettuce produced with different fertiliser treatments can be sufficiently different to be used for a double-blind study to study effects on a physiological response (BP). It showed that the high nitrate content caused an acute lowering of the systolic BP, although this was not a particular benefit for this study population. Therefore, we suggest to use the concept of HNLP versus LNHP lettuce (or similar), preferably together with a relevant control treatment, for long-term trials in relevant at risk populations, such as older individuals or hypertension patients. Such trials should focus on outcomes that are epidemiologically associated with nitrate-rich vegetables, and may determine whether NO_3_^−^ or phenolics are more important for the health benefits of this vegetable. If such a study shows any definitive outcome, its best treatment can be directly implemented as a functional food, without requiring the extensive safety assessments, etc. required for a novel food supplement or drug.

## Supplementary Information

Below is the link to the electronic supplementary material.

Supplementary file1 (PDF 2123 KB)

## Data Availability

Data and calculations described in the manuscript will be made available without restrictions upon request, pending specification of which data are requested and the purpose of their intended use.
