# Supplementary material for: Double-blind controlled dietary cross-over intervention with differentially fertilised intact lettuce leaves shows acute reduction in blood pressure in young adults, associated with faster uptake of nitrate than of phenolics
Source: Eur J Nutr. 2022 Jul 23;61(8):4191–203. doi: 10.1007/s00394-022-02961-5 (PMC9596532; doi:10.1007/s00394-022-02961-5)
Supplement: Supplementary file 1 — Supplementary file1 (PDF 2123 KB) [file 394_2022_2961_MOESM1_ESM.pdf]

Online Supporting material for: Placebo-controlled double-blind dietary cross-over intervention with differentially fertilised intact lettuce leaves shows acute reduction in blood pressure in young adults, associated with faster uptake of nitrate than of phenolics, published in European Journal of Nutrition, by Othman K. Qadir, Chris J. Seal, Ammar W. Ashor, Michele Tassotti, Pedro Mena, Daniele Del Rio, Mario Siervo and Kirsten Brandt, corresponding author kirsten.brandt@ncl.ac.uk, Newcastle University, UK.

Table S1. CONSORT checklist

Table S1 | CONSORT checklist of information to include when reporting randomised crossover trials			
Section/topic	Item No	Description	Page No*
Title†	1a	Identification as a randomised crossover trial in the title	1
Abstract†	1b	Specify a crossover design and report all information outlined in table 2	2
Introduction:			
Background‡	2a	Scientific background and explanation of rationale	4-6
Objectives‡	2b	Specific objectives or hypotheses	5-6
Methods:			
Trial design†	3a	Rationale for a crossover design. Description of the design features including allocation ratio, especially the number and duration of periods, duration of washout period, and consideration of carry over effect	6,7
Change from protocol‡	3b	Important changes to methods after trial commencement (such as eligibility criteria), with reasons	N/A
Participants‡	4a	Eligibility criteria for participants	6
Settings and location‡	4b	Settings and locations where the data were collected	6
Interventions†	5	The interventions with sufficient details to allow replication, including how and when they were actually administered	7
Outcomes‡	6a	Completely defined prespecified primary and secondary outcome measures, including how and when they were assessed	8,9
Changes to outcomes‡	6b	Any changes to trial outcomes after the trial commenced, with reasons	N/A
Sample size†	7a	How sample size was determined, accounting for within participant variability	10
Interim analyses and stopping guidelines‡	7b	When applicable, explanation of any interim analyses and stopping guidelines	N/A
Randomisation:			
Sequence generation‡	8a	Method used to generate the random allocation sequence	6
Sequence generation‡	8b	Type of randomisation; details of any restriction (such as blocking and block size)	6 (N/A)
Allocation concealment mechanism‡	9	Mechanism used to implement the random allocation sequence§ (such as sequentially numbered containers), describing any steps taken to conceal the sequence until interventions were assigned	6,7
Implementation†	10	Who generated the random allocation sequence,§ who enrolled participants, and who assigned participants to the sequence of interventions	6,7
Blinding‡	11a	If done, who was blinded after assignment to interventions (for example, participants, care providers, those assessing outcomes) and how	5,6
Similarity of interventions‡	11b	If relevant, description of the similarity of interventions	6, Fig. S1
Statistical methods†	12a	Statistical methods used to compare groups for primary and secondary outcomes which are appropriate for crossover design (that is, based on within participant comparison)	10,11
Additional analyses‡	12b	Methods for additional analyses, such as subgroup analyses and adjusted analyses	10,11

Table S1 | CONSORT checklist of information to include when reporting randomised crossover trials, continued			
Section/topic	Item No	Description	Page No*
Results			
Participant flow (a diagram is strongly recommended)†	13a	The numbers of participants who were randomly assigned, received intended treatment, and were analysed for the primary outcome, separately for each sequence and period	6, Fig.2
Losses and exclusions†	13b	No of participants excluded at each stage, with reasons, separately for each sequence and period	6
Recruitment‡	14a	Dates defining the periods of recruitment and follow-up	6,7
Trial end‡	14b	Why the trial ended or was stopped	N/A
Baseline data†	15	A table showing baseline demographic and clinical characteristics by sequence and period	Table 1
Numbers analysed†	16	Number of participants (denominator) included in each analysis and whether the analysis was by original assigned groups	6,10,11
Outcomes and estimation†	17a	For each primary and secondary outcome, results including estimated effect size and its precision (such as 95% confidence interval) should be based on within participant comparisons.¶ In addition, results for each intervention in each period are recommended	Fig.3-8, Fig S2 (not done by period, see p.10)
Binary outcomes‡	17b	For binary outcomes, presentation of both absolute and relative effect sizes is recommended	N/A
Ancillary analyses‡	18	Results of any other analyses performed, including subgroup analyses and adjusted analyses, distinguishing prespecified from exploratory	Fig.3-8 (all were exploratory, see p.10)
Harms†	19	Describe all important harms or untended effects in a way that accounts for the design (for specific guidance, see CONSORT for harms[32])	N/A
Discussion:			
Limitations†	20	Trial limitations, addressing sources of potential bias, imprecision, and if relevant, multiplicity of analyses. Consider potential carry over effects	15, 10
Generalisability‡	21	Generalisability (external validity, applicability) of the trial findings	13-16
Interpretation‡	22	Interpretation consistent with results, balancing benefits and harms, and considering other relevant evidence	13-16
Other information:			
Registration‡	23	Registration number and name of trial registry	2
Protocol‡	24	Where the full trial protocol can be accessed, if available	2
Funding‡	25	Sources of funding and other support (such as supply of drugs), role of funders	3

CONSORT=Consolidated Standards of Reporting Trials.
*Note: page numbers are optional depending on journal requirements. The page numbers shown here refer to the submitted manuscript.
†Modified original CONSORT item.
‡Unmodified CONSORT item.
§Random sequence here refers to a list of random orders, typically generated through a computer program. This should not be confused with the sequence of interventions in a randomised crossover trial, for example receiving intervention A before B for an individual trial participant.
¶A within participant comparison takes into account the correlation between measurements for each participant because they act as their own control, therefore measurements are not independent.

Table S2. Retention times and optimized SRM conditions for identification and quantification of caffeoylquinic acid-derived metabolites

No.	Compound	RT (min)	Parent ion (m/z)	S-lens	Quantifier		Qualifier	
					Product ion (m/z)	CE (V)	Product ion (m/z)	CE (V)
1	5-caffeoylquinic acid	3.42	353	83	191	20	135	36
2	4′-hydroxycinnamic acid-3′-glucuronide	3.33	355	98	179	21	135	37
3	4′-hydroxy-3′-methoxycinnamic acid	4.94	193	71	134	19	178	18
4	3′-methoxycinnamic acid-4′-glucuronide	3.18	369	93	193	18	134	39
5	3′-methoxycinnamic acid-4′-sulfate	4.36	273	92	193	18	178	28
6	3′-methoxycinnamic acid-4′-glycine	3.96	250	79	206	14	134	22
7	4′-methoxycinnamic acid-3′-glucuronide	3.90	369	93	193	18	178	32
8	3-(4′-hydroxyphenyl)propanoic acid-3′-sulfate	3.89	261	96	181	20	137	25
9	3-(3′-methoxyphenyl)propanoic acid-4′-sulfate	4.15	275	75	195	31	136	17

CE: collision energy

Experimental food

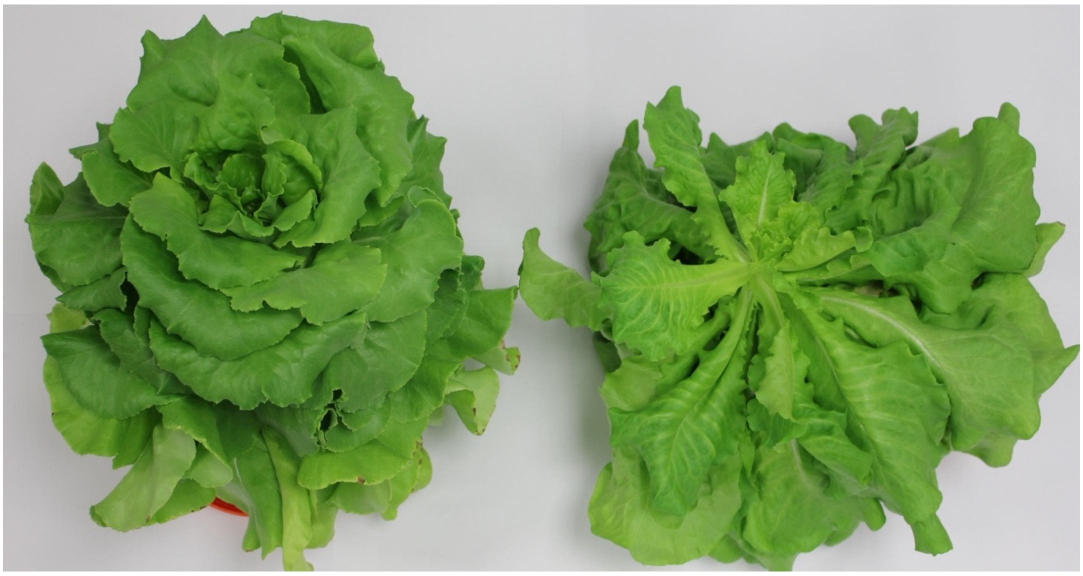

Fig. S1 Appearance of lettuce LNHP (left) and HNLP (right) [18] before separation of leaves and packaging

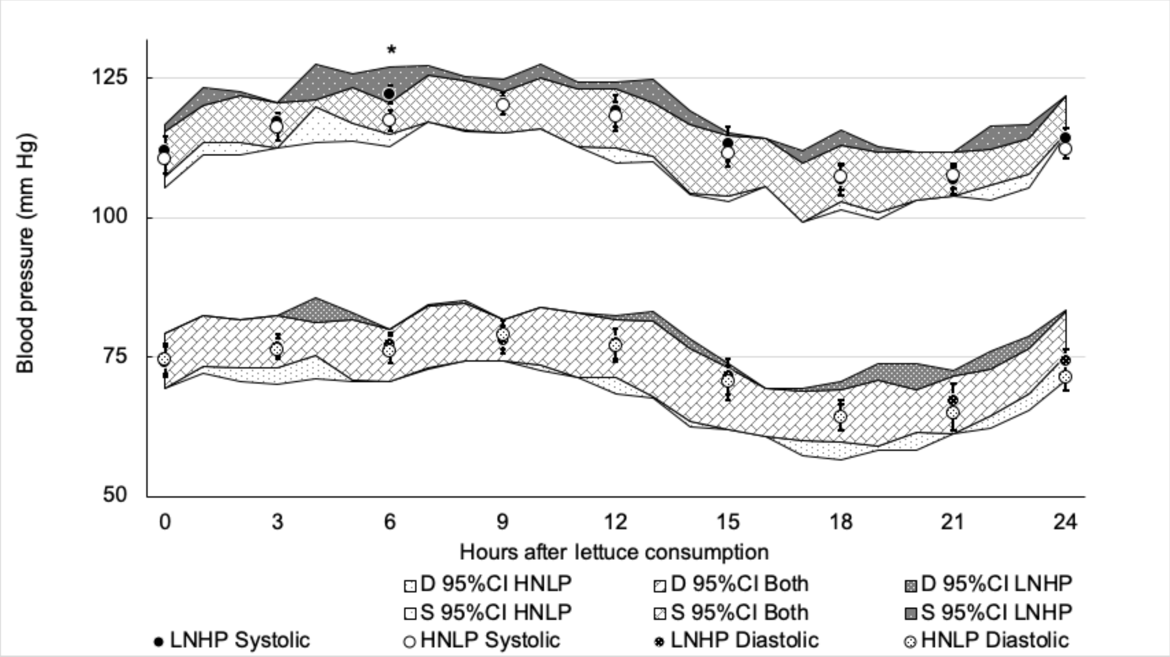

Fig. S2 The effect of HNLP and LNHP lettuce consumption on 24-hr ABPM systolic (S) and diastolic (D) BP showing 95% CI intervals. Average values for each 3-hour interval (values recorded up to 3 hours before) are shown as circles, SEM are shown as error bars, 95%CI is indicated by the patterned bands (hourly). The * indicates a significant difference between the treatments. (P=0.038).

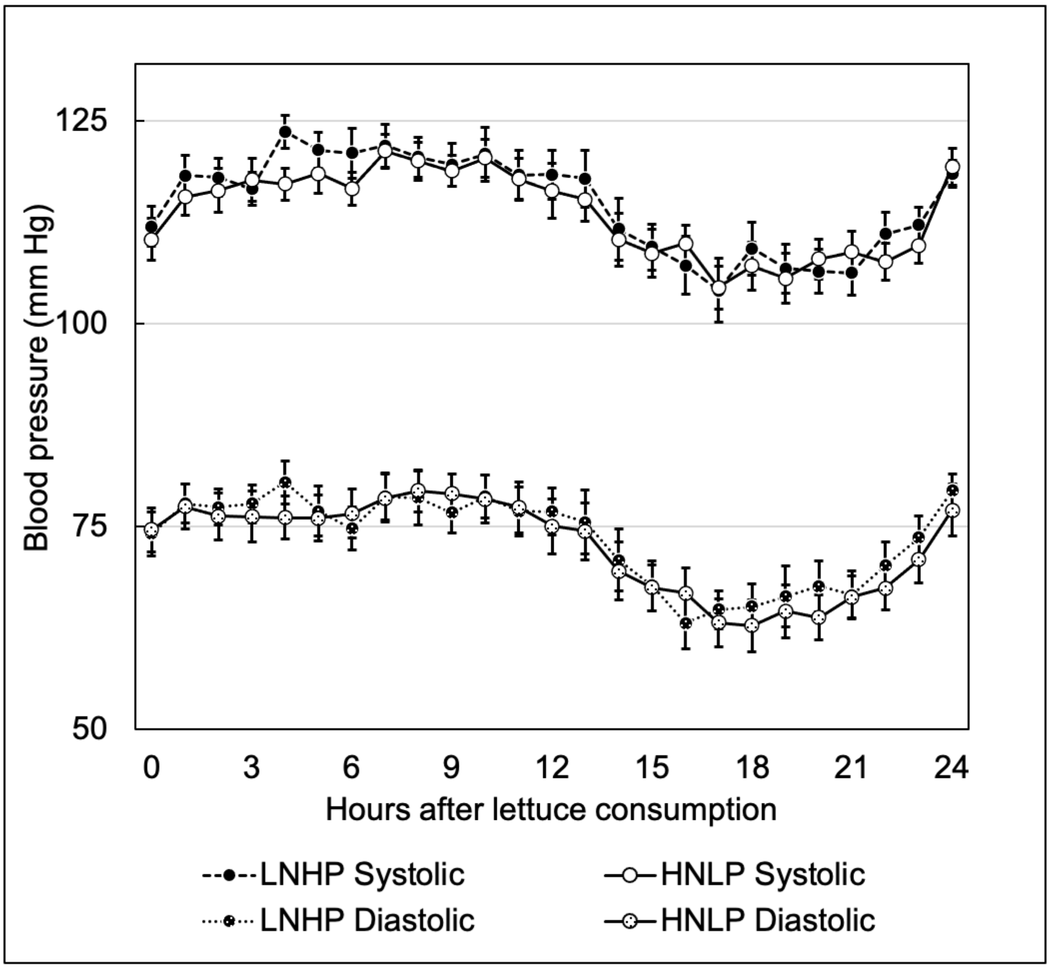

Fig. S3 The effect of HNLP and LNHP lettuce consumption on 24-hr ABPM systolic and diastolic BP. Average values for each 1-hour interval (values recorded up to 1 hour before) are shown as circles, SEM are shown as error bars.

Plasma phenolic compounds

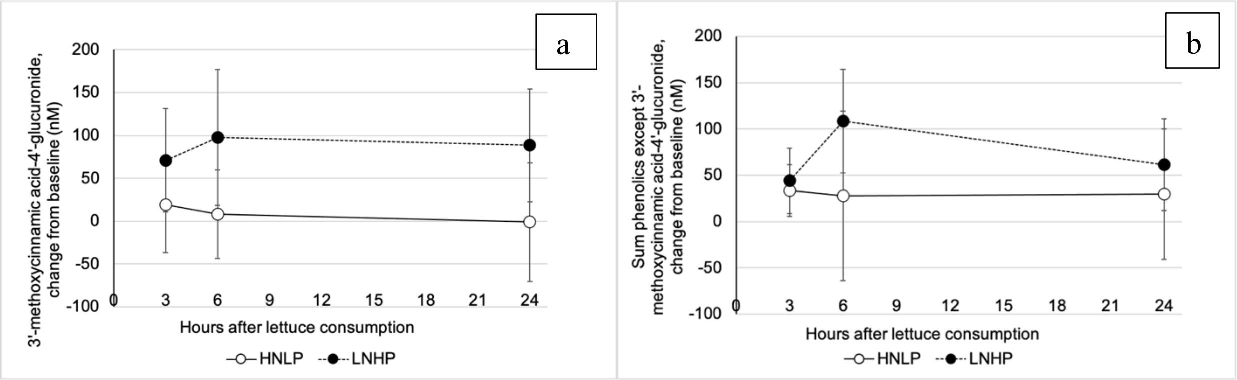

Fig. S4 The effect of HNLP and LNHP lettuce consumption on the change from baseline of the plasma concentrations of 3′-methoxycinnamic acid-4′-glucuronide (*aka* ferulic acid-4′-glucuronide) (a) and of the sum of all measured phenolic metabolites except 3′-methoxycinnamic acid-4′-glucuronide (b). Data expressed as mean ± 95% CI
